# Efficient Location Service for a Mobile Sink in Solar-Powered Wireless Sensor Networks

**DOI:** 10.3390/s19020272

**Published:** 2019-01-11

**Authors:** Minjae Kang, Ikjune Yoon, Dong Kun Noh

**Affiliations:** 1 Department of Electronic Engineering, Soongsil University, Seoul 06978, Korea; mjkang@ssu.ac.kr; 2 Department of Smart Systems Software, Soongsil University, Seoul 06978, Korea; ijyoon@ssu.ac.kr; 3 Department of Software Convergence, Soongsil University, Seoul 06978, Korea

**Keywords:** solar-powered, wireless sensor network, mobile sink, energy-aware, location service, scalable, blackout time, throughput, latency

## Abstract

By utilizing mobile sinks in wireless sensor networks (WSNs), WSNs can be deployed in more challenging environments that cannot connect with the Internet, such as those that are isolated or dangerous, and can also achieve a balanced energy consumption among sensors which leads to prolonging the network lifetime. However, an additional overhead is required to check the current location of the sink in order for a node to transmit data to the mobile sink, and the size of the overhead is proportional to that of the network. Meanwhile, WSNs composed of solar-powered nodes have recently been actively studied for the perpetual operation of a network. This study addresses both of these research topics simultaneously, and proposes a method to support an efficient location service for a mobile sink utilizing the surplus energy of a solar-powered WSN. In this scheme, nodes that have a sufficient energy budget can constitute rings, and the nodes belonging to these rings (which are called ring nodes) maintain up-to-date location information on the mobile sink node and serve this information to the other sensor nodes. Because each ring node only uses surplus energy to serve location information, this does not affect the performance of a node’s general operations (e.g., sensing, processing, and data delivery). Moreover, because multiple rings can exist simultaneously in the proposed scheme, the overhead for acquiring the position information of the sink can be significantly reduced, and also hardly increases even if the network size becomes larger.

## 1. Introduction

### 1.1. Motivation

A wireless sensor network (WSN) is a network composed of ultra-small, low-cost, and low-power sensor elements, and its wide-ranging fields of application include military surveillance, environmental monitoring, health care, home automation, and traffic control [1,2]. Because WSN nodes generally operate based on batteries, extensive research has been conducted to overcome the problem of limited energy resources. Therefore, researches have mainly focused on minimizing the amount of energy consumed by sensor nodes. However, in order to fundamentally solve the limited energy problem, research has actively been conducted in recent years on an energy-harvesting sensor node that collects and utilizes surrounding energy, such as environmental energy [3].

Energy-harvesting sensor nodes collect energy through the sun, pressure, wind, and temperature differences. Among these sources, solar energy is considered to be the optimal energy source for WSNs, owing to its energy density and periodicity. Because solar-powered sensors are charged periodically, their energy optimization goals differ from those of battery-based sensors. In other words, the goal of energy optimization for a solar-powered sensor is to maximize the energy utilization with consideration of its rechargeable battery capacity and energy-harvesting rate, while the goal for a battery-based sensor is to minimize its energy consumption.

On the other hand, applying a mobile sink to a WSN can solve the problem of energy imbalance between the nodes around the sink and the outer nodes, which typically occurs in a WSN with a fixed sink. However, if a sink moves on a random path (rather than a fixed one), a method of notifying each node of the sink’s location is required in order for those nodes to transmit data to the sink. The flooding scheme, which is the most naive method for informing all nodes of the location of the mobile sink, is simple and easy to implement, but its overhead, including the amount of packets, is huge, and increases exponentially with the network size owing to the redundancy of packets to be relayed. Moreover, the greater the mobility of the sink, the larger the overhead becomes.

Therefore, research aiming to reduce the cost of advertising the location information of mobile sinks has begun to be carried out. Among the investigated approaches, hierarchical methods using grids, clusters, and areas are the most actively studied [4]. In particular, area-based schemes are popular because they enjoy advantages in that they are easier and more efficient to construct than other schemes. However, they also suffer from the critical disadvantage of low scalability, which means that the efficiency can easily be affected by the size of the network (the number of nodes). Most area-based schemes suffer from significant performance degradation when the network size increases.

In this paper, we propose a more efficient and scalable mobile sink location management scheme for solar-powered WSNs.

### 1.2. Preliminaries

**Figure 1:**
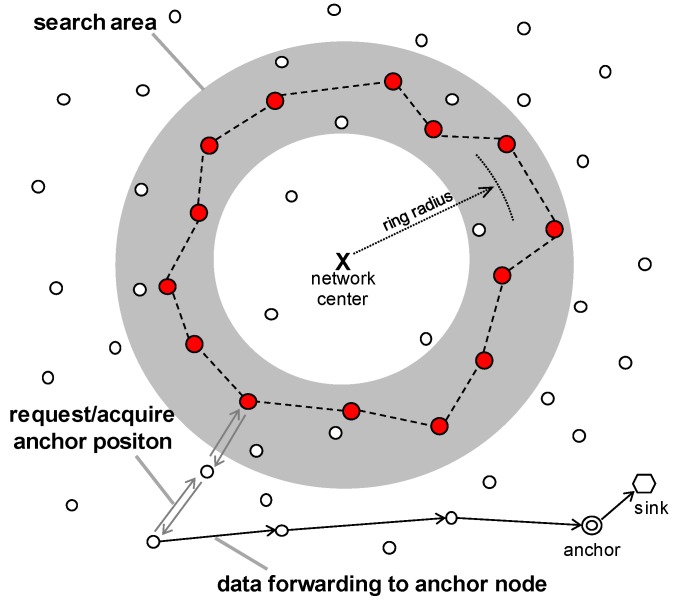
Overview of the ring routing [5] scheme.

This study concerns ring routing [5], which is a kind of area-based scheme. This scheme consists of a ring node, an anchor node, and a normal node. As shown in Figure 1, the gray search area separated by a ring radius distance from the center of the network is used to construct the ring. The nodes in this search area are connected to each other to form a ring, and only nodes belonging to the ring, which are called ring nodes, maintain the information on the location of the mobile sink.

When the energy of the ring node drops below a certain value, it selects a neighbor node that is farther from the ring center than itself and designates this as the next ring node. Then, the previous ring node switches to a normal node. Repeating this procedure will cause the ring to expand outside of the network. At the end of this expansion, most of the nodes have acted once as a ring node, which means that energy from all nodes is consumed in a balanced manner. After this time, the ring shrinks towards the center of the network. In this manner, the balance of energy is maintained while expanding and contracting the ring.

Meanwhile, when the mobile sink moves, a node close to it is selected as an anchor node. The anchor node operates as a temporary sink while the mobile sink is in its vicinity, collects the WSN data instead of a mobile sink, and finally transmits it to the mobile sink. Therefore, the location of the current anchor node (rather than the location of a mobile sink) is stored in the ring nodes.

The anchor node transmits its location information in the directions of both the center and outside of the network. This bidirectional transmission guarantees that the location data sent by the anchor node must be in contact with the ring (i.e., it must be sent to at least one of the ring nodes), regardless of the current position of the anchor node. The ring node that receives it transmits the location information to the neighbor ring nodes, and thus all the ring nodes can share the location information of the anchor node.

As shown in Figure 1, a sensor node that wants to transmit data to the sink requires the location information of the anchor node. To this end, it sends a packet requesting the location information of the anchor node in the direction of both the center and outside of the network, and receives a response packet with the location information. This bidirectional transmission ensures that the packet encounters one of the ring nodes. After receiving the location information of the anchor node, the sensory data is routed to the anchor node using the location-based routing method.

Although this scheme is highly efficient in relatively small WSNs, there can be a large variation in the overhead required for a node to reach the ring, depending on its position. Moreover, there is a critical problem in terms of scalability. As the size of the network increases, the number of hops required for nodes to acquire the location information also becomes larger. This increased overhead is reflected in the energy consumption of each node, and finally leads to a performance degradation.

### 1.3. Contribution

**Figure 2:**
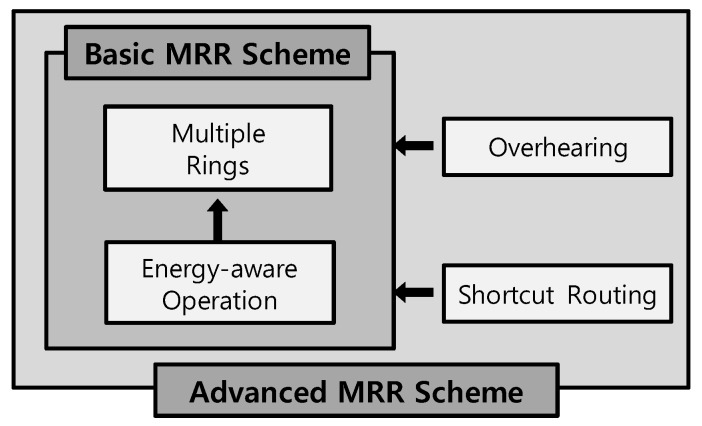
Sub-methods composing MRR scheme.

In this study, we propose a multiple-ring routing (MRR) scheme, which represents an efficient mobile sink location management system for solar-powered WSNs, tailored from the existing ring routing scheme. The MRR scheme is composed of several sub-methods as shown in Figure 2, all of which will be described in Section 3 and Section 4.

By maintaining more than one ring storing the recent location of the sink, it is possible to reduce both the size and variation of the overhead required by each node to locate the mobile sink. The proposed MRR scheme employs the energy model of a solar-powered node to utilize part of the harvested energy to construct a ring. Because this method only consumes surplus energy except for that required for typical operations of a sensor node, it does not degrade the performance of each node while maintaining multiple rings concurrently. In summary, the MRR scheme satisfies the following properties:

- The best utilization of harvested energy: each node can operate as a ring node only when it has surplus energy exceeding the amount of energy required for the general operation of a sensor node. This allows the MRR scheme to maximally exploit the periodically harvested solar energy.
- Enhancement of the data throughput: by efficiently maintaining multiple rings, the energy required for acquiring the location information of a sink node can be dramatically reduced. This conserved energy can be used to increase the amount of data collected at the sink.
- High scalability: owing to the multiple rings, the overhead required for acquiring the location information of the sink node hardly increases even if the network size becomes larger. This is a necessary requirement for large-scale WSN applications.
- Support for high mobility sinks: the transference of data to the fast sink node exhibits inevitably high failure rates. The MRR scheme prevents this problem to some extent by efficiently forwarding data to the recently moved sink node using the follow-up mechanism with shortcut routing.

Finally, Table 1 summarizes the contribution to the performance of WSNs for each of MRR scheme’s sub-methods shown in Figure 2.

This paper is organized as follows. In Section 2, we review previous research concerning both solar-powered WSNs and mobile sink location managers. In Section 3 and Section 4, we introduce our MRR scheme in detail, which represents an efficient mobile sink location manager for solar-powered WSNs. Section 5 verifies the performance of our MRR scheme by comparing it with the performances of other schemes, and we present our conclusions in Section 6.

## 2. Related Work

In this section, we describe the state-of-the-art concerning energy optimization for solar-powered WSNs and area-based hierarchical schemes for advertising the location of a mobile sink, both of which the proposed MRR scheme is related to.

### 2.1. Energy Optimization for Solar-Powered WSNs

Because battery-based sensor nodes suffer from a limited amount of energy, energy-harvesting sensor nodes are attracting attention in an effort to overcome this problem. These energy-harvesting sensor nodes collect energy periodically or sporadically, so that if they do not suffer hardware problems they can operate indefinitely. Among many environmental energy sources, solar energy is the most attractive for WSNs, because it has a higher power density than other energy sources, as shown in Table 2 [6], as well as being able to be harvested periodically. As a result, many solar-powered sensor nodes, such as Heliomote [7], have been developed.

One point to note is that solar energy is harvested periodically, but the amount of energy collected is not precisely predictable. Therefore, scheduling of the energy consumption rate is an essential requirement to utilize the harvested solar energy as effectively as possible. For example, if a node conserves its energy consumption during a daytime with a high energy density, then the harvested energy may exceed the capacity of the rechargeable battery. This excessive energy cannot be stored in the battery, and is inevitably discarded. Conversely, if a node maximizes its energy consumption during a daytime in order to minimize the amount of energy that must be discarded, it may not be able to operate normally during a nighttime with no energy collection. Therefore, research on scheduling the energy consumption rate for the best use of harvested energy in view of the battery capacity and harvesting rate is being conducted for solar-powered WSNs, while research on only minimizing the energy consumption at each node has been conducted for existing battery-based WSNs.

The first research issue that has been addressed concerning energy optimization for solar-powered WSNs is that of increasing the energy utilization in order to make a node operate stably while utilizing most of the harvested energy. Kansal et al. [8] introduced an energy model for solar-powered nodes that determines the energy bounds that allow a node to constantly survive. Melodia et al. [9] proposed a transmission energy consumption model considering the transmission distance, data length, path loss, and harvesting rate.

In addition, methods of predicting the amount of harvested solar energy have been studied. The amount of harvested energy must be accurately predicted and managed in order to ensure maximized utilization. However, trying to reach a high accuracy in prediction can be very difficult and expensive in terms of memory occupancy and complexity. Thus a trade-off between accuracy and computational overhead must be explored. Kansal et al. [7] introduced a harvested energy prediction schemes utilizing a moving average approach, which is called EWMA (exponentially weighted moving average). EWMA is the most popular algorithm and has inspired the development of many prediction approaches. It assumes that the energy generation profile at a particular time slot of the day exhibits similar behavior within the same slot to that of previous days. The fundamental principle of EWMA is to adapt to seasonal variations by maintaining the amount of harvestable energy in each time slot as a weighted average of energy available over a set of previous days. EWMA is very simple and effective, but it does not predict the amount of energy to reflect the short-term conditions such as weather. To handle the deficiencies of the EWMA, Piorno et al. [10] introduced WCMA (weather-conditioned moving average), which considers both current and past day solar conditions. Instead of considering only a weighted average as in EWMA, WCMA incorporates the energy harvested in the previous slot into the prediction equation. Inspired by WCMA, Bergonzini et al. [11] present the enhanced version of WCMA using a phase displacement regulator (PDR), which is able to reduce the peak errors of WCMA. It is meaningful that WCMA-based schemes apply weather factors to improve the accuracy of prediction, but it still cannot respond to factors such as temporal climate change or shadows. To address this problem, Cammarano et al. [12] proposed the Pro-Energy (profile energy prediction model), which can facilitate more precise predictions using long-term and short-term data. Pro-Energy considers the amount of energy harvested in the previous slot as in WCMA. However, a matrix, *E*, maintaining the energy harvested in the past of *D* days is also derived and maintained. The distinctive feature of Pro-Energy is that the most similar day to the current day in terms of energy generation is obtained from the *E* matrix. Therefore, a combination of energy observed in the previous slot and the energy from the most similar day contribute to predicting the current energy. Similarly, Kosunalp [13] proposed a solar energy prediction algorithm with Q-learning (QL-SEP). The distinctive feature of QL-SEP is that not only past days’ observations but also the current weather or shadow conditions are considered for prediction.

Recently, methods for allocating energy to each time slot have been studied for a balanced use of time-varying solar energy. Noh et al. [14] proposed a scheme to allocate the appropriate amount of energy to time slots considering historical data according to the time and weather. However, the energy allocation is not tightly coupled with the flow control, and their scheme does not explicitly include the stochastic behavior of energy harvesting in energy model. Zhang et al. [15] proposed a control scheme for the rate of data gathering by dynamically allocating energy to each time slot. Yoon et al. [16] proposed an adaptive data aggregation and compression method to improve energy utilization. In this scheme, each node in a WSN periodically determines its amount of allocated energy taking into account its residual energy and its likely acquisition and consumption of energy. According to the allocated energy, it is determined whether the sensed data is aggregated with data from other nodes, compressed, and transmitted. This scheme also does not include stochastic behavior, and it can be used only for the limited applications because it can only be used for periodic data collection.

From the point of view of energy optimization, our main goal in this study is to maximize the energy utilization by controlling the energy consumption rate, and to employ the surplus energy to maintain multiple rings.

### 2.2. Location Management for a Mobile Sink

A WSN with a mobile sink distributes the energy consumption of the intermediate nodes, which relay data to the sink node. As a result, the energy imbalance problems that occur between the nodes around a fixed sink and the outer nodes can be resolved to some extent. However, in a WSN with a mobile sink that traverses a random path (rather than traversing a fixed path), additional energy is required to track the location of the mobile sink [17].

Therefore, many schemes for locating the sink node using less energy have been studied. Among these, hierarchical schemes, which divide nodes into several hierarchies and manage the location of a mobile sink only in a specific hierarchy, have been actively studied. These hierarchical schemes are categorized into grid-based [18,19,20], cluster-based [21,22,23], and area-based approaches [5,24,25], according to how the hierarchy is divided. In the case of the grid-based approach, problems such as isolated nodes occur. Furthermore, clustering-based approaches suffer from the problem of a high overhead related to the construction of clusters. On the other hand, in the area-based approach the above problems are relatively slight, because the distances between nodes are considered when dividing hierarchies. The following are well-known schemes based on the area-based approach.

- LBDD (Line-Based Data Dissemination) [24]: In LBDD, nodes between two straight lines in the center of the network are called inline nodes, and these share the location information of a mobile sink. A source node that wants to deliver data to a sink node simply transmits data to these inline nodes, and the inline node that has received the data routes it to the mobile sink.
- Railroad [25]: RailRoad is a data dissemination architecture for large-scale WSNs. The RailRoad system proactively exploits a virtual infrastructure called a Rail, which is an area where all the metadata of events is stored. There is only one Rail in a network, and it acts as a rendezvous area for events and queries. The Rail is placed in the middle area of the field, so that every node can easily access it. Once a query is issued, it circulates around the Rail and searches relevant data stored in the Rail. When relevant metadata is found, the source node of the data transmits the corresponding data to the sink node that issued the query.

We note that LBDD should set positionally balanced lines so as to prevent traffic from being shifted to one of them, and RailRoad should set the Rail direction to meet the node containing the metadata in order to avoid a performance degradation. Unlike these approaches, the ring routing scheme referred by this paper has no special considerations when setting the area, and it is possible to evenly utilize the energy of each node by extending and shrinking the ring repeatedly. However, as previously explained, there is a large variation in the overhead required for each node to locate the mobile sink according to the position of the node, and the overhead for acquiring location information may become exponentially larger as the network area increases.

## 3. Proposed MRR Scheme

MRR scheme proposed in this research improves the data throughput and scalability of a WSN by constructing multiple rings using surplus solar energy. In addition, because mobile sinks can change their positions even during data forwarding, the greater the mobility of a sink the greater the probability that data forwarding will fail. MRR can also be effectively applied to a mobile sink that frequently changes its location.

### 3.1. Overview of the MRR Scheme

**Figure 3:**
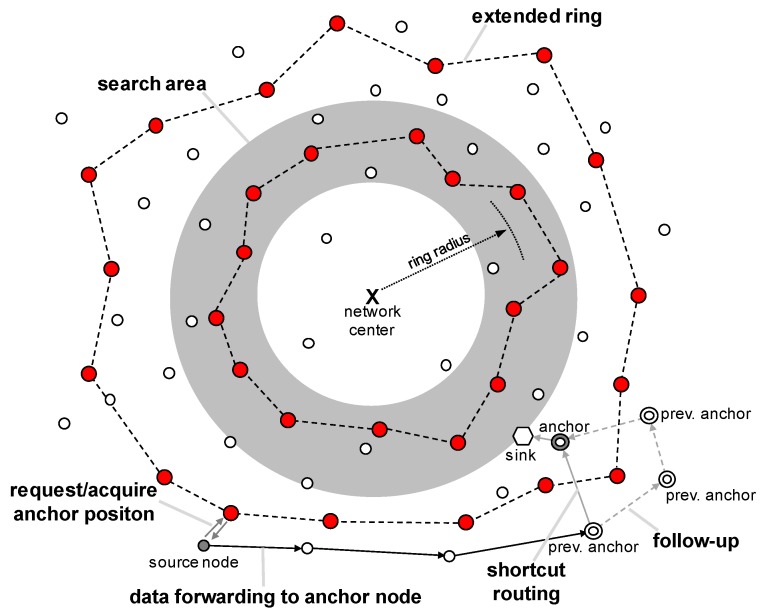
Overview of the MRR scheme.

Figure 3 depicts the overall operation of MRR. First, the sink chooses an anchor node that is sufficiently close to reach and has sufficient energy. Then, the selected anchor node notifies its location to the ring nodes. When a source node wants to transmit data to the sink, it first requests the location information of the anchor from a ring node and receives a response. Then, the data is routed to the anchor node using the location-based routing scheme. When the anchor node receives data from a node, it transfers it to the sink if the sink is still close to it. If the sink has already moved and is not close to it, then it performs a follow-up mechanism so as to pass the data to the current anchor node with the shortest path.

Meanwhile, the management of multiple rings is performed in three stages. First, a ring is created in the search area. Then this ring continuously extends outward from the network center. In the last step, when the ring that has been extended reaches the end of the network it is disassembled. Note that multiple rings can exist at the same time, because a ring is created in the search area whenever possible.

Note that a node in our MRR scheme requires geographical information for itself, its neighboring nodes, and the network center, because MRR is based on an area-based sink-locating scheme and location-based routing scheme.

### 3.2. Energy Model

This section describes an energy threshold to determine whether the current energy level of a node is sufficient for it to operate as a ring node. The ideal energy model for the solar-powered system should be constructed considering the solar energy harvesting rate and energy consumption rate of the system. The former depends on the location, season, and weather, while the latter depends on the data-sensing rate, data-transmission rate, and duty-cycle. Unfortunately, it is difficult to predict these factors precisely. Therefore, Yang et al. [26] proposed an effective and simple energy model that is independent of these factors, which is summarized in this section.

Let $P_{\mathrm{charge}}$ be the average rate at which solar energy is obtained by a node and $P_{\mathrm{sys}}$ be the average rate at which energy is consumed by the system. Each of these is estimated using moving averages when the system is harvesting and consuming energy. If the residual amount of energy in node $n_{i}$ is $E_{\mathrm{residual}}\left(i\right)$, then the expected time until the battery is fully charged can be expressed as follows:

(1) $$T_{\mathrm{full}}\left(E_{\mathrm{residual}}\left(i\right)\right)\;=\;\frac{C\left(i\right)\;-\;E_{\mathrm{residual}}\left(i\right)}{P_{\mathrm{charge}}\left(i\right)\;-\;P_{\mathrm{sys}}\left(i\right)},$$

where C(i) is the battery capacity of node $n_{i}$. An important issue is that the average energy consumption rate should be lower than the average solar energy harvest rate, because the battery is only charged when $P_{\mathrm{charge}}\left(i\right)\;>\;P_{\mathrm{sys}}\left(i\right)$. Fortunately, although $P_{\mathrm{charge}}\left(i\right)$ is not controllable, $P_{\mathrm{sys}}\left(i\right)$ can be roughly controlled by adjusting the duty-cycle or sensing rate control. Therefore, we can satisfy the inequality $P_{\mathrm{charge}}\left(i\right)\;>\;P_{\mathrm{sys}}\left(i\right)$ by a suitable system configuration.

Solar energy can only be harvested during the daytime and varies from day to day. However, to avoid blackout until the battery is fully charged, the amount of energy remaining in the battery must satisfy the following:

(2) $$E_{\mathrm{residual}}\left(i\right)\;\geq \;P_{\mathrm{sys}}\left(i\right)\;\cdot \;T_{\mathrm{full}}\left(E_{\mathrm{residual}}\left(i\right)\right).$$

This is true even in the worst-case scenario where solar energy is only harvested for a moment. We can obtain $E_{\mathrm{residual}}\left(i\right)\;\geq \;\left(P_{\mathrm{sys}}\left(i\right)\;/\;P_{\mathrm{charge}}\left(i\right)\right)\;\cdot \;C\left(i\right)$ by solving Equations (1) and (2). That is, if there is at least as much energy as $P_{\mathrm{sys}}\left(i\right)\;/\;P_{\mathrm{charge}}\left(i\right)\;\cdot \;C\left(i\right)$ in the battery, then an unexpected blackout will not occur. This value is called the energy threshold $E_{\mathrm{threshold}}\left(i\right)$, which is formulated as follows:

(3) $$E_{\mathrm{threshold}}\left(i\right)\;=\;\frac{P_{\mathrm{sys}}\left(i\right)}{P_{\mathrm{charge}}\left(i\right)}\;\cdot \;C\left(i\right)$$

In summary, if $E_{\mathrm{residual}}\left(i\right)$ becomes greater than $E_{\mathrm{threshold}}\left(i\right)$, this means that there is sufficient energy to perform the basic operations of the node (such as sensing and transfer), so that it may perform other additional tasks. Therefore, a node satisfying $E_{\mathrm{residual}}\left(i\right)\;\geq \;E_{\mathrm{threshold}}\left(i\right)$ becomes candidate for a ring node in our proposed scheme. Conversely, a node that satisfies $E_{\mathrm{residual}}\left(i\right)\;<\;E_{\mathrm{threshold}}\left(i\right)$ is changed to a normal node.

As described until now, our MRR scheme allows each node to act as a ring node or a general node adaptively according to the amount of energy in an energy-rechargeable environment such as solar-powered WSNs. However, in general battery-based WSNs, each node only consumes energy without any charging process, so the amount of energy does not fluctuate but monotonously decreases. Therefore, energy-adaptive operations of MRR scheme cannot be performed. This is the reason why MRR scheme cannot be expected to improve the performance of a battery-based WSN to the extent that it is applied to a solar-powered WSN. At least, however, if MRR scheme is applied to a battery-based WSN, it will perform the same operation as the existing ring routing scheme that keeps only one ring.

### 3.3. Management of Multiple Rings

This section describes the management of rings. Each ring is repeatedly operated in the order of creation, expansion, and release, and one or more rings can exist at the same time depending on the energy status.

#### 3.3.1. Ring Construction

**Figure 4:**
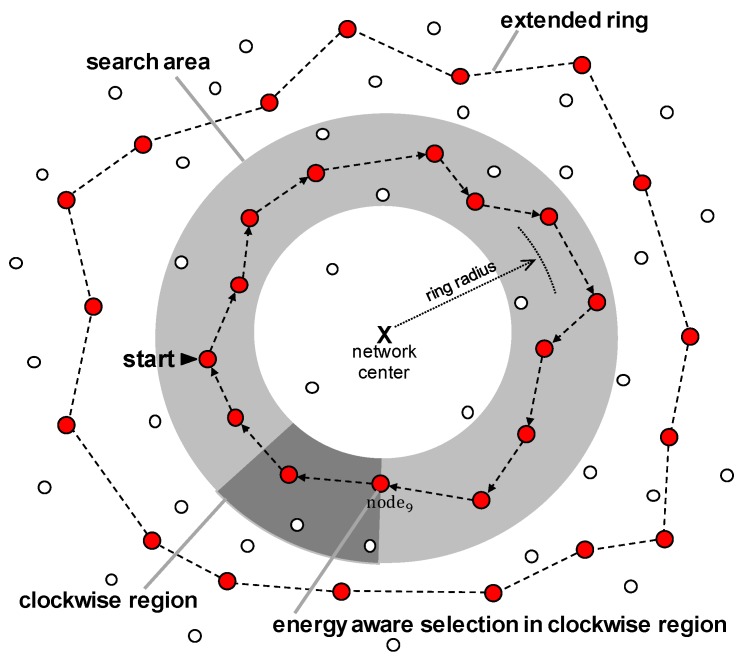
Ring construction of MRR scheme.

In the proposed MRR method, one or more rings are created to provide the location of a sink node. The creation of a ring is performed in a fixed search area. Then, the created ring will be gradually extended, and when the ring moves out of the search area completely, the construct a new ring in the search area is attempted. As time passes, the probability that a new ring will be constructed in the search area increases, because $E_{\mathrm{residual}}\left(i\right)$ gradually increases. Figure 4 illustrates this ring construction process.

The selection of a ring node to construct a ring is performed using Equation (4). First, one of the nodes in a search area whose residual energy satisfies Equation (4) starts to construct the ring by finding and connecting to a node in a search area that also satisfies Equation (4) among the neighbors in the left region with respect to itself when looking at the center of the network. For convenience, we refer to this side of region as the clockwise (CW) region of that node and the opposite side as the counter-clockwise (CCW) region. The dark gray region in Figure 4 represents the CW region relative to ${\mathrm{node}}_{9}$.

(4) $$E_{\mathrm{residual}}\left(i\right)\geq E_{\mathrm{threshold}}\left(i\right)$$

If the ring is not established at the current period, then the construction of a ring will be attempted again in the following periods. As explained in Section 3.2, $E_{\mathrm{residual}}\left(i\right)$ becomes larger as time progresses, because $P_{\mathrm{sys}}\left(i\right)$ is always smaller than $P_{\mathrm{charge}}\left(i\right)$ when node *i* only performs its typical operations (not operating as a ring node). Thus, as time progresses the probability of constructing a ring in the search area also increases.

It should be noted that there must be at least one ring in the network, because a node should be able to know the location of the mobile sink to send data to it. For this purpose, the MRR scheme designates several nodes in the search area as initiation nodes, which are responsible for the quick creation of a ring. If these initiation nodes notice that the last ring is about to disappear (the method of how initiation nodes learn this information will be discussed in Section 3.3.3), they attempt to construct a ring through the method of selecting the node with the highest energy among the neighbors as a ring node, although Equation (4) is not satisfied. This situation may happen in practical scenarios where ring-node candidates in a search area are scarce due to the environment of that area which lacks solar energy. Multiple rings can hardly exist because a ring is hard to be created in these environments. However, even in this harsh situation, the MRR scheme works in the same way as the existing ring routing scheme by making compulsorily at least one ring in any case. This means that the worst performance of MRR is same as the performance of ring routing scheme which was proven to have better performance than flooding scheme based on mesh structure.

#### 3.3.2. Ring Extension

A ring node consumes energy to function not only as a sensor node, but also as a location information service, so that the residual energy is rapidly reduced, and finally the energy state no longer satisfies Equation (4) after some period of time passes. This means that there is not sufficient residual energy to act as a ring node. Because this ring node needs to reduce its energy consumption to avoid being blacked out, it changes to a normal mode that performs typical operations as a sensor node, and a new ring node is selected to replace it.

**Figure 5:**
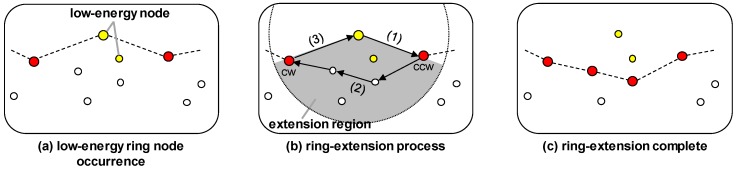
Ring extension of MRR scheme.

Figure 5 illustrates the process of extending the ring. If the energy state of the ring node no longer satisfies Equation (4), so that it becomes difficult to act as a ring node, as illustrated by the yellow ring node in Figure 5a, then the ring is extended as shown in Figure 5b, which is explained step-by-step as follows:

1. To extend the ring, the low-energy ring node announces the ring extension to the neighboring ring node in its CCW region (called the CCW ring node). At this time, the information on both the neighboring nodes in the extension region and the neighboring ring node in the CW region (called the CW ring node) are both provided. The extension region refers to the region swept out in the clockwise direction between the CCW ring node and the CW ring node, which constitutes a region outside of the ring within the transmission range of the node.
2. The CCW ring node that has been notified of the ring extension finds the nodes satisfying Equation (4) among the candidate nodes in the extension region. Of course, there may or may not be any corresponding nodes. If at least one node is found, then the CCW ring node determines the most energy-efficient path containing these nodes from itself to the CW ring node, and the nodes on this path are designated as new ring nodes. On the other hand, if there are no corresponding nodes then the CCW ring node chooses the node with the most residual energy among the candidate nodes as a new ring node. This is possible because the CCW ring node receives all the information of nodes in extension region and the CW ring node. Finally, the CCW ring node sends a control packet to the CW ring node through these new ring nodes.
3. When receiving the control packet, the CW ring node notifies the low-energy ring node that the ring extension is completed. Then, the low-energy ring node changes its role to a basic node.

Finally, Figure 5c illustrates the result after the ring has been extended.

#### 3.3.3. Release of Ring

**Figure 6:**
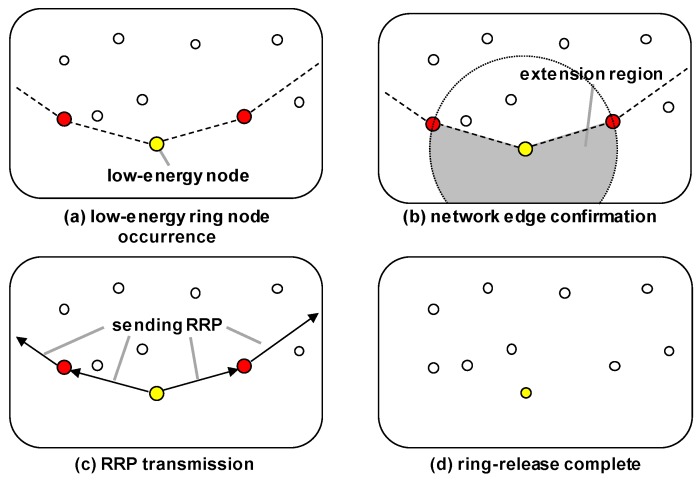
Release of ring in the MRR scheme.

If the energy state of a node does not satisfy Equation (4), as shown in Figure 6a, then the ring extension process is performed. However, if the node is at the edge of the network, as shown in Figure 6b, then the ring release is performed, because the ring cannot be extended anymore. As shown in Figure 6c, when the ring is released the node that initiates the ring release process sends a ring release packet (RRP) to its neighbor ring nodes on both sides of the same ring, and the nodes that receive this relay it to their neighbor ring nodes. After this each ring node changes its role to be a normal node, and when all the ring nodes have received an RRP, the ring release operation is completed, as shown in Figure 6d.

If less energy is harvested or the network size is small, then there may not be another ring at the time that one is released. In this case, the ring releasement process should not be carried out, because the network must have at least one ring to deliver data to the mobile sink. Therefore, a ring node that wants to be released must send a query packet requesting the location of the mobile sink node in order to confirm whether at least one ring exists in the network. If there is no another ring, then a control packet is sent to the initiation nodes in the search area in order for them to create new ring, as explained in the final paragraph of Section 3.3.1, and the ring releasement process is postponed until the new ring has been constructed.

### 3.4. Data Routing to the Mobile Sink with Multiple Rings

This section explains how a source node forwards sensory data to the mobile sink node using the anchor nodes and multiple rings.

#### 3.4.1. Anchor Node Selection

The mobile sink node receives data using anchor nodes for reliable data reception, because an anchor node is fixed at one point while the location of the mobile sink node frequently changes. The sink node selects the node that has the most energy among those in its communication range as an anchor node, and the anchor node advertises its location to the ring nodes. The previous anchor node can learn the location of the new one, and this information is useful to utilize for shortcut routing (as described in Section 4.1 in further detail).

**Figure 7:**
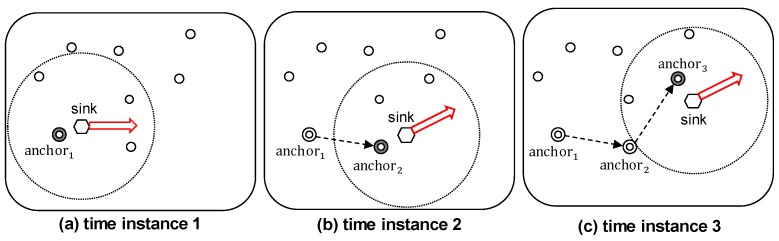
Anchor Node Change According to the Location of the Sink Node.

Figure 7 illustrates the change of anchor node when the sink is continuously moving. During the time that the anchor node is still within the communication range of the mobile sink, as shown in Figure 7a, the anchor node does not change. In contrast, when the mobile sink moves out of communication range, as shown in Figure 7b, it must select a new anchor node. If the sink moves out of the range of ${\mathrm{anchor}}_{2}$, as shown in Figure 7c, then ${\mathrm{anchor}}_{3}$ is selected as a new anchor node, and its information is transmitted to the previous anchor node ${\mathrm{anchor}}_{2}$.

#### 3.4.2. Advertising Anchor Node’s Location

**Figure 8:**
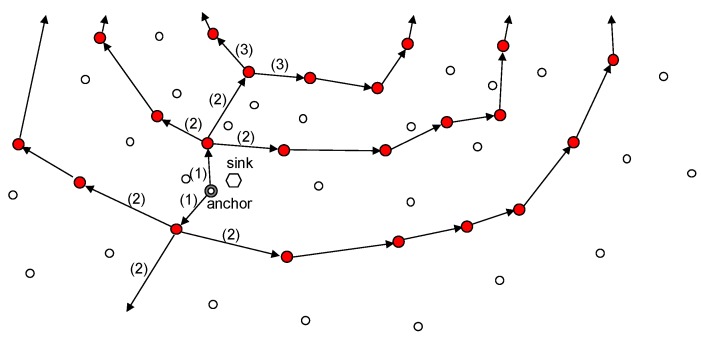
Advertisement of an anchor node’s position.

After an anchor node is selected, it should advertise its location to all the ring nodes. Figure 8 illustrates this process. As shown in the figure, first the anchor node transmits its location information both towards the network center and in the opposite direction to propagate its location information to the ring nodes. Then, each ring node receives the information packet works as follows:

1. If a ring node receives the information packet from other node instead of neighboring CW or CCW ring nodes, it transmits the packet to its neighboring ring nodes on both the CW and CCW sides. Simultaneously, it also forwards the packet in the direction in which the packet was originally travelling (i.e., the center or outside of the network).
2. If a ring node receives the information packet from one of its neighboring ring nodes, it transmits data to the opposite-side ring node.
3. If a ring node receives the same information packet from both of two neighboring ring nodes, it sends a sink location packet no longer because this means that all ring nodes belonging to that ring have received this packet.

The problem of packet loss should be addressed when sink’s location information is advertised. The primary reasons of packet loss are interference, low-power, and congestion. However, in WSN, the throughput of data is relatively small compared with that of general network, so packet loss due to congestion is negligible. This is the reason why the transport layer has not been studied extensively in WSN. Also, because the ring nodes are generally energy-rich nodes, it does not need to consider packet loss due to low-power. In MRR, therefore, ARQ in a link layer is adopted to solve the loss of location information packet occurred mainly by interference. The ARQ protocol guarantees hop-to-hop packet delivery, which gradually allows all ring nodes to receive new location information packet.

#### 3.4.3. Delivery of Data to the Sink Node

**Figure 9:**
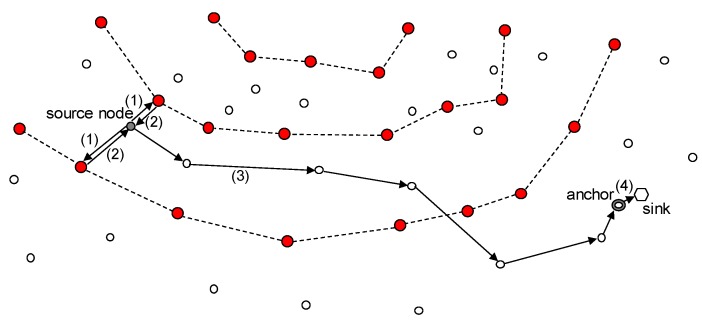
Request of anchor node’s position and data forwarding.

A source node requires location information for the anchor node for data transmission. As shown in Figure 9, the process of obtaining the location information for the anchor and delivering the data to the sink node proceeds as follows:

1. The source node sends a location information request packet in the direction of the network center and the opposite direction in order to obtain the location information. This ensures that at least one ring node receives the packet.
2. The source node can obtain the location information for the anchor node by receiving the response packet from a ring node.
3. It then sends data to the anchor node using the location-based routing scheme.
4. The anchor node transmits the received data to the nearby sink.

If the anchor node determines that there is no sink node within its communication range, this means that the sink node has moved, and so it performs the follow-up mechanism to transfer the data to the newly located sink node. This is discussed in detail in Section 4.1.

## 4. Advanced MRR Scheme

We add two advanced features to the MRR scheme explained thus far, which are called shortcut routing and advertisement overhearing, respectively. For convenience, the MRR scheme with these two features is referred to as advanced MRR.

### 4.1. Follow-Up Mechanism with Shortcut Routing

**Figure 10:**
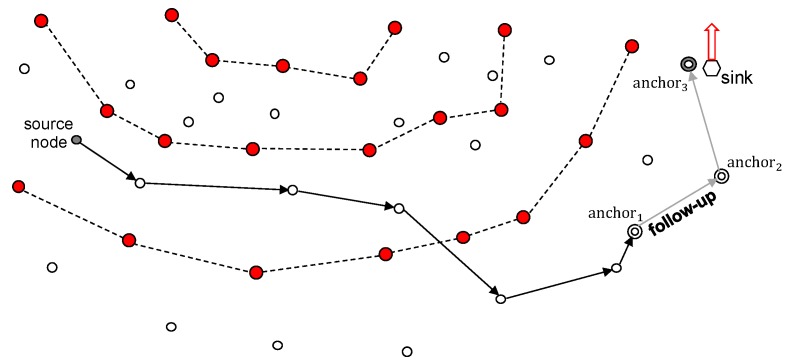
Follow-up mechanism.

A mobile sink with high mobility can move while data is being transferred from a source node to the anchor node, which can lead to a loss of data. To prevent this, MRR employs the follow-up mechanism illustrated in Figure 10. As described in Section 3.4.3, the previous anchor node stores the location of the successor. Based on this location information, it is possible to forward data to the new anchor node even though there is no sink close to the anchor node that received the data.

**Figure 11:**
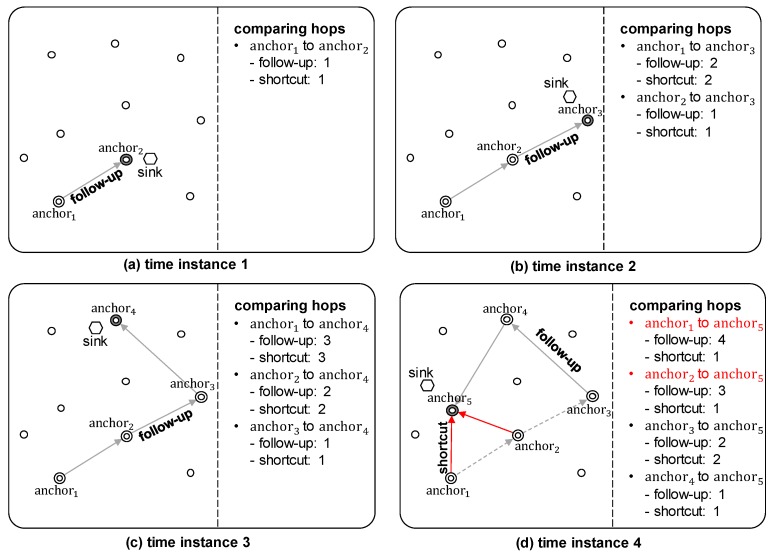
Shortcut routing.

However, this simple follow-up method is inefficient, because in general the direction of movement of the sink is random, as shown in Figure 11. To improve the efficiency, we propose an advanced MRR scheme using shortcut routing. In shortcut routing, the sink node stores the locations of anchor nodes whenever it chooses a new anchor, and uses this information to calculate $H_{\mathrm{sc}}^{i}$, which is the number of hops on the shortest path from each previous anchor node *i* to the current anchor. Shortcut routing is performed if $H_{\mathrm{sc}}^{i}$ satisfies the following equation:

(5) $$H_{\mathrm{fu}}^{i}>H_{\mathrm{sc}}^{i}\times \alpha ,$$

where $H_{\mathrm{fu}}^{i}$ is the number of hops from the anchor node *i* to the current anchor node, and $H_{\mathrm{sc}}^{i}$ is the number of hops on the shortest path. α is a weighting factor, with a default value of 2, and this increases in proportion to the speed of the mobile sink node. The reason for α being at least 2 is because the previous anchor node must receive a request packet for shortcut routing from the sink node to perform shortcut routing, which results in an overhead. Figure 11 shows an example of determining whether to employ shortcut routing when the previous anchor nodes (${\mathrm{anchor}}_{1}$∼${\mathrm{anchor}}_{4}$) send data to the current anchor node (${\mathrm{anchor}}_{5}$). ${\mathrm{anchor}}_{1}$ and ${\mathrm{anchor}}_{2}$ perform shortcut routing, because they satisfy Equation (5), while ${\mathrm{anchor}}_{3}$ and ${\mathrm{anchor}}_{4}$ perform the follow-up mechanism.

The advanced MRR scheme with this shortcut routing can reduce the unnecessary data transmission overhead of the follow-up mechanism, thereby reducing the total amount of consumed energy. In particular, this is efficient in the case that the sink node moves quickly and randomly.

### 4.2. Overhearing Anchor’s Position

We incorporate the feature of an overhearing method into the MRR scheme, which implicitly obtains location information to reduce the communication overhead compared with the explicit method of acquiring the location information for the anchor node explained in Section 3.4.3.

**Figure 12:**
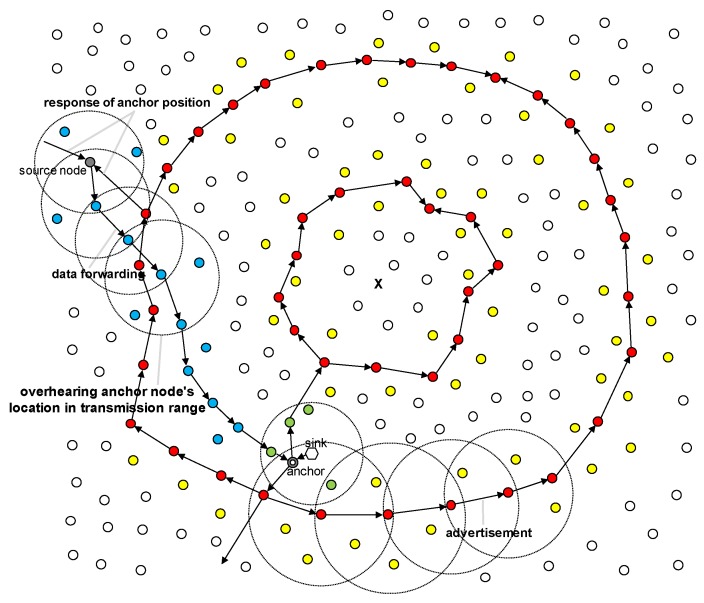
Overhearing anchor’s position.

In WSNs, sensor nodes can generally overhear packets even if they are not the destination nodes, because they communicate with each other by broadcasting. At this time, if a packet has location information for the anchor node, then the location information can be obtained by reading the data without dropping the packet. Therefore, if packets have location information for the anchor node, then nodes can obtain this location information by overhearing. MRR operations where overhearing can be performed are described as follows:

- Anchor node selection: When the sink selects an anchor node, nodes close to the sink node can obtain location information for the anchor node by overhearing packets transmitted between the sink node and anchor node (the green node in Figure 12).
- Advertisement of the anchor node’s location: When the anchor node informs the ring of its location information and when this location information is shared between the ring nodes, nodes on the transmission path can obtain the location information (yellow nodes in Figure 12).
- Data forwarding: When the source node receives location information from the ring for data transmission, nodes on the path between the source and ring node and their neighbors can obtain the location information. In addition, when the source node sends data to the anchor node, nodes on the path and their neighbors can also obtain the location information (blue nodes in Figure 12).

The overhearing efficiency depends on the number of rings and the size of the field. However, in most cases this method can considerably reduce the overhead of the location information queries.

## 5. Performance Evaluation

We tried to verify the performance of proposed MRR and advanced MRR by comparing them with previous flooding scheme and original ring routing scheme, using SolarCastalia [27] which is a simulation tool designed for the solar energy-harvesting WSN.

### 5.1. Simulation Environments

Table 3 lists the important parameters used in our simulation. The amount of energy consumed (communication, sensing, and peripherals) and solar energy harvested for each node in this simulation represent actual values measured using Texas Instruments EZ430-RF2500-she [28,29]. The ratio of movement to stopping was set to 1:3 based on the average movement of a person [30]. This is similar to a soldier’s movement when a WSN application is employed in warfare.

### 5.2. Scalability

**Figure 13:**
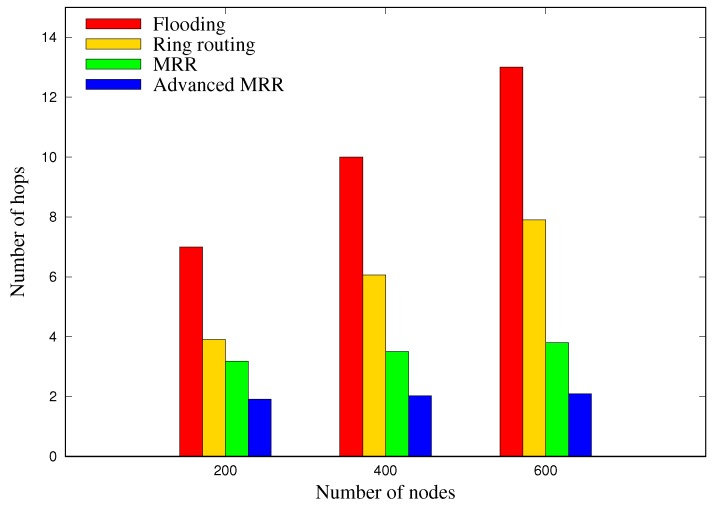
Number of hops required for each node to obtain the sink position for sending the data, which is the average value of 50 runs in each network size.

Figure 13 shows the average number of hops for each node to obtain location information of sink node from the ring, according to the various network sizes. The flooding scheme, in which the sink node broadcasts its location to all nodes whenever it moves, exhibits the highest increase in the overhead, with up to about 13 hops at 600 nodes. The number of hops for our MRR scheme is lower by 22%, 42%, and 56% in comparison with the original ring routing method when the number of nodes is 200, 400, and 600 respectively. This is because the MRR scheme creates and utilizes multiple rings unlike the original ring routing scheme as shown in Table 4. Note that as explained before, maintaining multiple rings in MRR scheme does not reduce the functionality of each node because it only uses extra energy to maintain multiple rings, which would be proved in Section 5.3. Finally, it can be seen that the advanced MRR scheme, which reduces the number of anchor node location requests through overhearing, reduces the number of hops by approximately 46% compared with the MRR scheme. Additionally, it can be confirmed that unlike the flooding or ring routing, the overheads (number of hops in Figure 13) required to acquire the location information of sink node, in both of MRR and advanced MRR, are hardly affected by the network size. This result demonstrates that the proposed MRR and advanced MRR are much more scalable than other schemes.

### 5.3. Blackout Nodes

**Figure 14:**
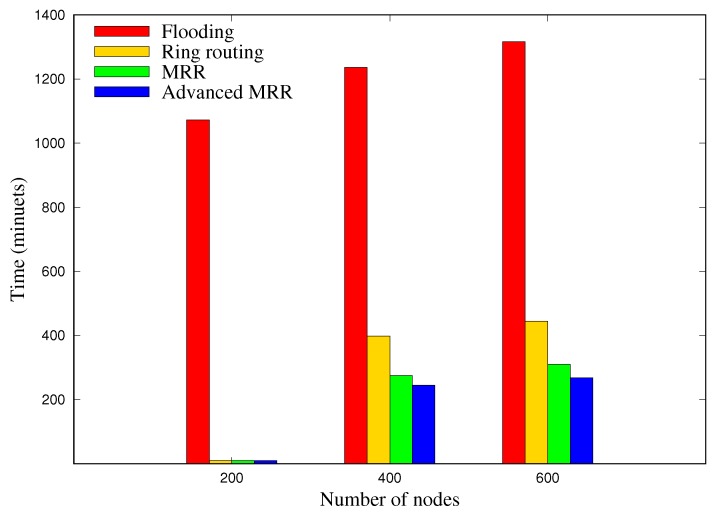
Expected blackout time for each node for 30 days, which is the average value of 50 runs in each network size.

Figure 14 depicts the average blackout time of each node for 30 days, according to the network size. Overall, the blackout time of flooding scheme is extremely high compared with the other schemes since it works in a way that consumes a lot of energy, and the advanced MRR shows the least blackout time, regardless of the network size. In the case of 600 nodes, for example, the number of blackout nodes in MRR was reduced by 78% and 31% compared to flooding and ring routing, respectively, and that of advanced MRR was 9% lower than MRR. These results prove that maintaining multiple rings in MRR and advanced MRR schemes does not degrade the functionality of each node because they only use extra energy to maintain multiple rings. We also infer that the advanced MRR schemes show the fewest blackout nodes due to the overhearing and shortcut routing.

**Figure 15:**
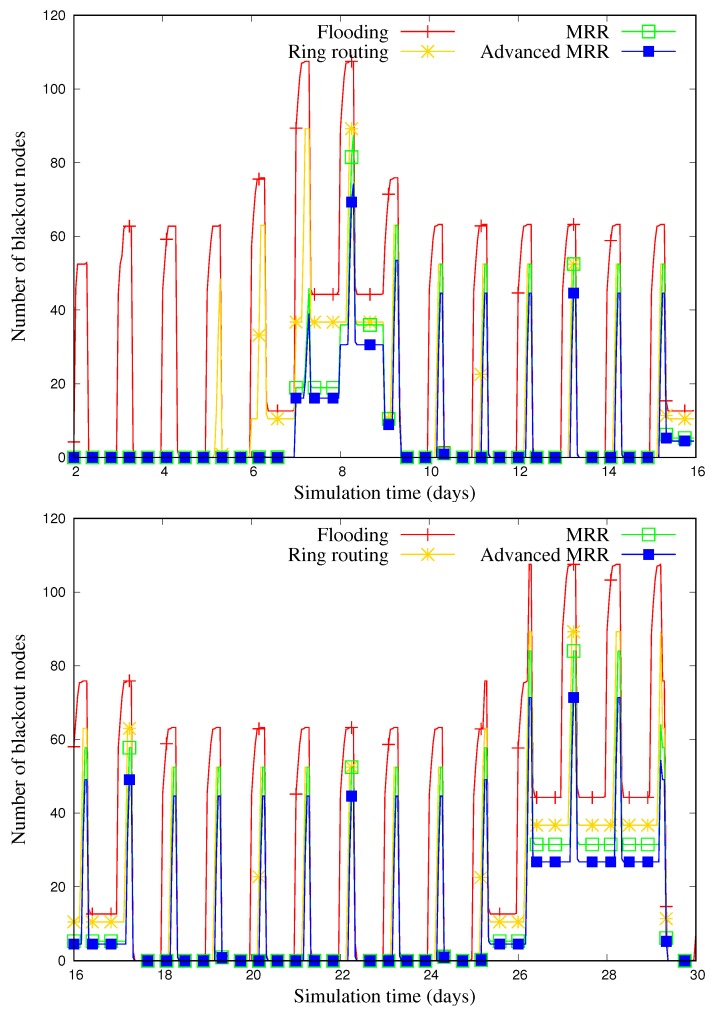
Tracing the number of blackout nodes on a sample run out of 50 runs when the number of nodes is 600 (6th∼9th day: rainy, 16th∼17th day: cloudy, 25th∼29th day: rainy, and others: sunny).

Figure 15 illustrates the change in the number of blackout nodes over time for 30 days, when the number of nodes is 600. Overall, for all of schemes, it is shown that the number of blackout nodes increases when the weather is rainy or cloudy and decreases when it is sunny. Although the value varies depending on the weather, we can find that the MRR and advanced MRR are less affected by the weather than other schemes because of their energy-aware operation.

### 5.4. Amount of Collected Data

**Figure 16:**
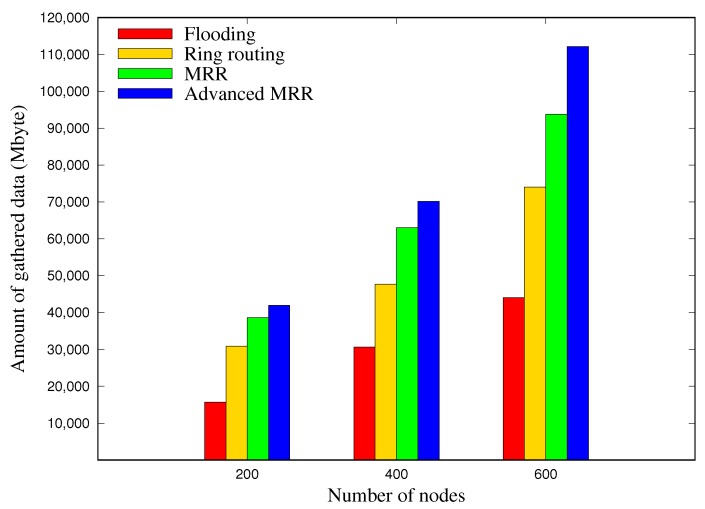
Amount of gathered data by the sink node, which is the average value of 50 runs in each network size.

Figure 16 shows the amount of data gathered at the sink node for 30 days, according to the network size. In the case of 400 nodes, the amount of gathered data in MRR was greater by 116% and 38% compared with the flooding and ring routing schemes, respectively, and in the advanced MRR scheme this was 9% greater than for MRR. In the case of 600 nodes, the advanced MRR scheme increased the amount of gathered data by 144%, 57%, and 18% compared with other three respective schemes. We note that the difference became larger as the network size increased.

**Figure 17:**
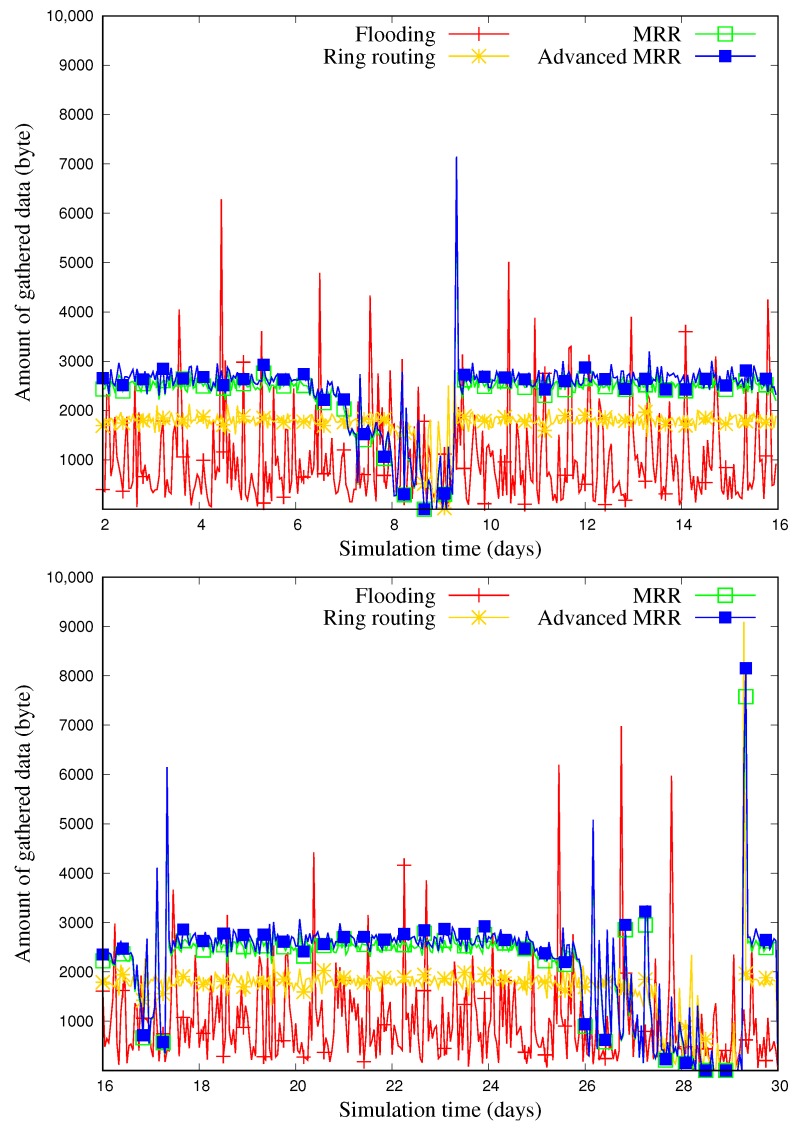
Tracing the amount of gathered data on a sample run out of 50 runs when the number of nodes is 600 (6th∼9th day: rainy, 16th∼17th day: cloudy, 25th∼29th day: rainy, and others: sunny).

Figure 17 shows the change in the amount of gathered data at the sink node over time when the number of nodes was 600. Similar to the number of blackout nodes in Figure 15, the amount of gathered data for every scheme fluctuates according the weather condition. However, it can be confirmed that our MRR and advanced MRR show the higher performance generally with any circumstance, even though they maintain multiple rings.

## 6. Conclusions

For solar-powered WSNs, research towards utilizing harvested energy as effectively as possible has been required, because energy is continuously harvested. Meanwhile, for a WSN employing a mobile sink node it is necessary to study how to efficiently advertise the location of the mobile sink. This study addresses both these topics simultaneously. In the proposed scheme, the harvested energy is utilized to maintain multiple rings in order to efficiently provide the location information of the mobile sink. The proposed scheme generates and maintains multiple rings using only surplus energy, so that nodes can efficiently obtain the location information of the sink node. This can lead to an increase in the data throughput of the sink node. Furthermore, unlike the original ring routing scheme our scheme exhibits good scalability, which means that the performance is not degraded even when the network size increases.

## Figures and Tables

**Table 1:** Contribution by sub-methods of MRR scheme.

	Contribution	Increasing Solar Energy Utilization	Decreasing Blackout Time	Increasing Data Throughput	Enhancing Network Scalability	Supporting Sink’s High Mobility
Sub-Methods						
Energy-aware operation		⋁				
Multiple rings			⋁	⋁	⋁	
Overhearing method			⋁	⋁		
Shortcut routing method						⋁

**Table 2:** Performance of energy harvesters [6].

Energy Harvester	Power Density	
	Indoor/Human Condition	Outdoor/Industrial Condition
Solar panel	100 $\mu W/{\mathrm{cm}}^{2}\;@\;10\;W/m^{2}$ irradiance	10 $\mathrm{mW}/{\mathrm{cm}}^{2}\;@\;1000\;W/m^{2}$ irradiance
Wind turbine generator	35 $\mu W/{\mathrm{cm}}^{2}\;@\;<1\;m/s$ wind speed	3.5 $\mathrm{mW}/{\mathrm{cm}}^{2}\;@\;8.4\;m/s$ wind speed
Thermoelectric generator	100 $\mu W/{\mathrm{cm}}^{2}\;@\;5{\;}^{\circ }C$ gradient	3.5 $\mathrm{mW}/{\mathrm{cm}}^{2}\;@\;30{\;}^{\circ }C$ gradient
Electromagnetic generator	4 $\mu W/{\mathrm{cm}}^{3}\;@\;$ human motion-Hz	800 $\mu W/{\mathrm{cm}}^{3}\;@\;$ machine-kHz

**Table 3:** Experimental environments.

Parameter	Value
Number of nodes	200∼600
Size of topology	4900∼14,884 $m^{2}$
Deployment	Random
Weather	Randomly selected (sunny, cloudy, or rainy)
Average energy harvesting rate	33.5 J/day
Transmission range	10 m
Energy consumption rate for Data TX	0.16704 μJ/byte
Energy consumption rate for Data RX	0.18912 μJ/byte
Baud rate	250 kbps
Sensing data size	32 bytes
Battery capacity	200 mAh
Avg. speed of mobile sink	1 km/h
Moving direction of mobile sink	Random
simulation time	30 days

**Table 4:** Average number of rings per network size.

	Scheme	Flooding	Ring Routing	MRR	Advanced MRR
# of Nodes					
200		N/A	1	1.556	1.648
400		N/A	1	2.408	2.466
600		N/A	1	4.060	4.211
